# Editorial: Advances in cardiac anesthesiology and cardiopulmonary bypass for cardiac surgery and interventions

**DOI:** 10.3389/fmed.2025.1649654

**Published:** 2025-07-18

**Authors:** Mohamed R. El Tahan, Osama Abou-Arab, Emmanuel Besnier

**Affiliations:** ^1^Cardiothoracic Anaesthesia and Surgical Intensive Care, Mansoura University, Mansoura, Egypt; ^2^Cardiothoracic Anaesthesia, Imam Abdulrahman Bin Faisal University (formerly University of Dammam), Dammam, Saudi Arabia; ^3^Department of Anesthesia and Critical Care, CHU Amiens, Amiens, France; ^4^Laboratoire MP3CV, UR 7517, Université Picardie Jules Verne, Amiens, France; ^5^Département d'Anesthésie-Réanimation, CHU Charles Nicolle, Rouen, France; ^6^Faculté de Médecine et de Pharmacie, Université de Rouen, Rouen, France; ^7^INSERM U1096 - EnVI, Rouen, France; ^8^Intensive Care Committee, Société Française d'Anesthésie et de Réanimation (SFAR), Paris, France

**Keywords:** anesthesia, cardiac, cardiopulmonary bypass, myocardial protection, dexmedetomidine, lung ultrasonography, biomarkers

## Introduction

Cardiac anesthesiology and perfusion medicine have undergone rapid transformation in recent years, reflecting the expanding complexity of surgical procedures and the growing need to individualize perioperative management. The interplay between anesthetic techniques, myocardial protection, extracorporeal circulation, and organ preservation has become increasingly important in defining short- and long-term outcomes in cardiac surgery.

In light of these evolving challenges and opportunities, we launched this Research Topic, “*Advances in cardiac anesthesiology and cardiopulmonary bypass for cardiac surgery and interventions*,” to provide a focused academic platform that brings together current perspectives, innovative techniques, and practical insights from around the world. The aim was to highlight perioperative strategies that optimize outcomes during cardiac surgical interventions—ranging from myocardial protection and monitoring to sedation strategies and organ-specific risk stratification.

**Figure 1:**
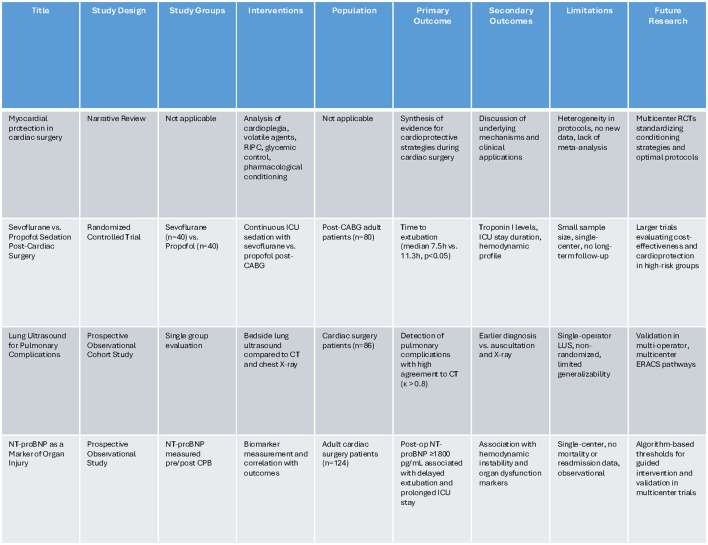
A table compares four studies on cardiac surgery advancements. Columns include Title, Study Design, Study Groups, Interventions, Population, Primary Outcome, Secondary Outcomes, Limitations, and Future Research. Rows cover topics like myocardial protection, sedation techniques, lung ultrasound for complications, and NT-proBNP as a marker of organ injury. Each study is detailed with specific methodologies, outcomes, and suggestions for future research including larger trials and validation efforts. Limitations include small sample sizes and generalizability issues.

The Research Topic received 15 submissions, and after a thorough peer review, four articles were accepted for publication. These studies reflect the diversity of research in our field and collectively provide timely contributions to clinical and research practice. Below, we summarize their key findings and outline future research directions. Figure 1 shows the strengths and limitations of these four studies.

## Conclusion and future research directives

This inaugural Research Topic underscores the evolving landscape of cardiac anesthesia and perfusion medicine. Each contribution highlights a critical component of perioperative care—ranging from myocardial protection to organ-specific diagnostics and sedation optimization. Notably, the articles emphasize the transition from empirical practice to precision-guided strategies based on objective markers and early diagnostic tools.

Moving forward, we encourage the cardiac anesthesia community to prioritize the following:

- Multicenter collaborations that bridge cardiovascular anesthesiology and surgical teams,
- Pragmatic trials that assess long-term outcomes and implementation feasibility,
- Integration of non-invasive monitoring (e.g., ultrasound, biomarkers) into enhanced recovery protocols,
- Translational research that connects molecular insights with bedside interventions.

We sincerely thank all authors, peer reviewers, and editorial staff for their contributions to this Research Topic. We hope this Research Topic serves as a springboard for continued innovation in perioperative care and a stimulus for broader engagement across surgical, perfusion, and critical care disciplines.

